# Short and Long-Term Physiological Responses of Male and Female Rats to Two Dietary Levels of Pre-Gelatinized Maca (Lepidium Peruvianum Chacon)

**Published:** 2006-02

**Authors:** H. O. Meissner, B. Kedzia, P. M. Mrozikiewicz, A. Mscisz

**Affiliations:** 1 *Faculty of Health Studies, Charles Sturt University & Therapeutic Research, TTD International Pty Ltd, GPO Box 4792, Sydney 2001, Australia;*; 2 *Research Institute of Medicinal Plants, 61-707 Poznan, 27 Libelta St., Poland*

**Keywords:** Maca (Lepidium peruvianum), toxicity, hormones, male-female rats, pharmacology

## Abstract

**Objective:**

The aim of this study is to identify physiological responses of male and female rats to either a short- or long-term administration of two doses of Maca (Lepidium peruvianum) and observe relationships which may exist between groups of hormones and effects mediated by them.

**Design:**

The effect of pre-gelatinized (extruded) organic Maca powder (Maca-GO) was studied on Sprague-Dowley male and female rats (1:1 ratio) receiving two dietary levels of Maca-GO (0.75 g/kg and 7.5 g/kg body weight) and assessed against control during 28 and 90 day laboratory trials on 30 and 60 rats respectively. Blood morphology, biochemistry (hormones, lipids and minerals) and histology of internal organs were determined. Homogenates of skeletal muscles and bones of rats were also analyzed.

**Results:**

Maca-GO has low toxicity (LD=7.5 g/kg) and appears to be safe for short-term and extended use as dietary supplement or as a component of functional dietary and therapeutic preparations. There were different responses of male and female rats to different levels of Maca-GO administered during a short- and a longer-term periodl. When administered at higher dose for extended period of time (90 days), Maca-GO acted as a toner of hormonal processes in adult female rats at increased progesterone and a steady estradiol level, without affecting levels of blood FSH, LH and TSH.

**Conclusions:**

Obtained results justify further clinical research on use of Maca-GO in sportsmen, physically-active people of both sexes and peri-menopausal women to clarify mechanisms underlaying physiological mode of action of Maca-GO validaet in clinical study on humans. Substantial decrease in blood cortisol levels in a short- and longer-term trial and simultaneous tendency to lower blood ACTH, may indicate antidepressive effect of Maca-GO, which together with reduction in body weight, lowering triglycerides in blood plasma and increasing calcium and phosphorus deposition in bone and muscle tissues is worthy consideration in potential application to women at both, pre- and postmenopausal stage.

## INTRODUCTION

Maca (Lepidium peruvianum Chacon), which grows in the high Andean plateaus of Peru and was described in more details previously (1, 2), has been traditionally used by native Peruvians as both food and medicine. In recent years, variety of commercial preparations based on Maca have been introduced on markets around the world, classified as over the counter (OTC) dietary supplements or as dietetic food supplements for special medicinal applications to enhance physical, physiological and psychological performance. Many aspects of traditionally-recognised biological activity of Maca are not well documented (3, 4, 5) and most of them are based on testimonials and anecdotal evidence, which, although supported by authentic and reputable reference source, lacks scientifically-accepted credibility.

Some of traditional applications of Maca have been in recent years experimentally proven (1, 6, 7, 8, 9), mainly in regards to fertility and energy enhancing properties in humans, improving libido and sexual functioning, hormone balancing and alleviating menopausal discomfort. Results of studies conducted so far, may suggest that action of Maca relies on plant sterols, which act as chemical triggers to help the body itself produce a higher level of hormones appropriate to the age and gender of person taking it (10), the fact already established by Chacon (11) in her pioneer study on Maca back in early 1960s. Chacon concluded that there are four alkaloids in the Maca root, responsible for fertility effects on the ovaries and testes of the rats, which were measurable within 72 hours of administration to the animals. She has concluded that since Maca contains no plant hormones, therefore the alkaloids were acting on the hypothalamus-pituitary gland axis, which may explain why both male and female rats were afflicted in a gender-appropriate manner. This may also explain why the effects of Maca in humans are not limited to ovaries and testes, but through acting on the adrenals, gives a feeling of greater energy and vitality and through this possibly affecting the pancreas and thyroid as well.

Current commercial interest in Maca turned into its energizing and revitalizing properties and its successful use in alleviating menopausal symptoms. In a pilot clinical study on early-postmenopausal women volunteers (1) it has been shown that Maca can be of value in the treatment of menopausal syndrome. However, although hormonal profiles and standardised subjective assessment of symptoms experienced by participants (Greene’s Menopausal Index) were presented, it was not possible to assess a mode of action and a role of Maca in balancing hormones along the hypothalamo-pituitary-ovarian axis. In the same study, noticeable differences were observed in results obtained during shorter (two months) and a longer-term (eight months) administration of Maca preparation. Also, Muller (4, 5) noted that the response of menopausal women to Maca is dose-dependent and should be taken into consideration in determining the level of daily dose, either for hormone-balancing effect at lower Maca doses, or as opposed to stimulating Maca mode of action, which may be achieved at high levels of administration.

In this study, using a model laboratory experimental design, an attempt has been made to demonstrate the effect of a short (28 days) and a longer-term effect (90 days) of administration of two levels of Maca (0.75 g/kg and 7.5 g/kg body weight respectively) on blood morphology, biochemistry, histo-pathology of internal organs and chemical analyses of muscle and bone tissues in male and female rats. Simultaneously, a toxicity (LD50) of Maca-GO preparation as used in this study was determined.

## MATERIAL AND METHODS

### Maca (Lepidium peruvianum Chacon)

The plant species was described in details in monographs by Chacon (11) and Obregon (12) as well as in the catalogue of the flowering plants and gymnosperms of Peru (13). The depositions of Maca plant identified as Lepidium peruvianum Chacon, after formal authentication by San Marcos Herbarium in Lima have been deposited in Herbarium of Medicinal Plants in Australia (Lismore) and in Poland (Poznan) for reference purpose and to verify authentcity of the material used in internationally-coordinated laboratory and clinical study and, if required, to authenticate analytically commercial preparations marketed under the name of Maca.

Maca roots, used in the present study were harvested in Junin area (Central Andean Region of Peru between 4200 m and 4500 m altitude) and represented typical distribution of three main ecotypes (out of 13 known): black, yellow and purple/red roots observed in this cultivation area - averaging to approximately 16%, 48% and 9% respectively. After some three month drying at high altitude (on the plantation site), according to traditionally used system of dehydration, considered superior to oven dried method currently used in commercial “modern” operations in Peru (Obregon, 2001 and Chacon 2003 - personal communications), dried Maca roots selected for this study were transported to a processing plant at the National Institute of Agricultural Research (NIAR), National Agricultural University La Molina in Lima (Peru) after previous attestation of its organic status, and its authentication by Dr Gloria Chacon as cultivated Maca Lepidium peruvianum Chacon which represent the same plant species, which she used in her pioneering work on Maca published back in 1961 (10).

After cleaning (washing under pressure) and cutting into pieces, dried hypocotyls of Maca were re-hydrated prior to being exposed to a gelatinization process comprising of exposure to a short-term elevated pressure under moist conditions (a proprietary extrusion process), followed by drying and pulverizing. Such treatment of Maca, without any chemicals used in the process, resulted in the final powdered product (Maca-GO) achieving increased density and through pre-gelatinization of a starch component in the product (not less than 98% according to BRI Laboratory assay, Sydney, Australia), expected to promote its easier digestion and bio-availability.

Composition of the pre-gelatinized Maca-GO powder (batch TTD-ZMP-20100351) as per analyses conducted in NIAR in Lima, Peru and in Analytical Laboratory of the Research Institute of Medicinal Plants in Poznan is given in Table 1.

**Table 1:** Composition of Pre-Gelatinized Maca-GO (*Lepidium peruvianum* Chacon)

No.	Specification	Unit per 100 g of product	Pre-Gelatinized Maca Root Powder (Maca-GO)

1	Energy value	kJ/kcal	1,235/295
2	Moisture	g	5.8
3	Ash	g	4.9
4	Crude Protein	g	11.7
5	Ether Extract	g	4.1
6	Carbohydrates Total	g	73.5
7	Available Carbohydrates	g	52.0
8	Dietary Fiber	g	21.5
9	Vitamin C	mg	659.3
10	Thiamine	μg	167.1
11	Calcium	mg	318
12	Phosphorus	mg	352
13	Sodium	mg	52
14	Potassium	mg	1373
15	Glucosinolates as Synigrine	mg	200
16	Unsaponified fraction	% oil fraction	16
17	Campestral	% unsuponified	7.8
18	Sigmasterol	% unsuponified	4.1
19	β-sitosterol	% unsuponified	24.2
20	Arginine	mg	300
21	Gelatinization Index^a^	%	98.5

a Degree of gelatinization of starch obtained as a result of extrusion process. Assay conducted using the method by the BRI Laboratory, Sydney, Australia.

### Animals and treatment

The study was carried out at the Research Institute of Medicinal Plants in Poznan between December 2003 and June 2004. The studies were performed on 90 Sprague-Dowley rats sourced from the Toxicology Department, Medical University in Poznan: 45, nine week old females weighing 240 g to 250 g and 45, twelve weeks males weighting 340 to 350 g at the beginning of the experiment. Males and females were kept separately (5 rats per cage), under standard animal laboratory conditions at temperature 22°C (+/-2°C) and relative humidity 55% (+/-5%). The animals had free access to standard laboratory diet (pellets-Muligran) and to tap water in their cages. All the experiments were conducted in compliance with relevant OECD standards (Recommendation No. 408) regarding procedures for testing toxicity of unknown toxic substances on rodents (14) and conforming to the relevant Polish Law (35/03).

Experiment was conducted under a standard laboratory model approved by a Bioethics Committee for Animal Experimentation of the Research Institute of Medicinal Plants (RIMP) in Poznan. Applied in this study experimental protocol was adopted from a standard OECD method used in determination of toxicity of unknown products (14). Maca-GO was administered to restrained rats (positioned in a vertical position) by intubation of 0.75 g/kg or 7.5 g/kg suspended in 15 ml water. Control group was intubated with corresponding volume of water. Intubations were carried out for 5 days each week during either 28 or 90 days trial.

Short-term study (28 days): In this Trial (I) - animals were randomly assigned to three groups, each of 10 animals (50% male and 50% female), two groups receiving Maca-GO at the level of 0.75g/kg and 7.5g/kg respectively and the third being a control group.

Sub-chronic study protocol (90 days): In this Trial (II) animals were randomly assigned to three groups of 20 animals each (50% male and 50% female), two groups receiving Maca-GO at the level of 0.75 g/kg and 7.5 g/kg respectively and the third being a control group. On completion of the each Trial, all rats were weighed and blood samples were taken for analyses under thiopental narcosis. In trial II, after blood sampling, the following internal organs were dissected: liver, pancreas, spleen, kidneys and gonads for histo-pathological examination of internal organs. Also, both legs were dissected for analysis of homogenates of skeletal muscles and bones for proximate analyses extended for calcium and phosphorus determination.

Toxicity (LD_50_) was determined according to Litchfield-Wilcoxon method modified according to Roth (1961). Blood which was collected at the end of each Trial (between 09:00 hr and 11:00 hr from male and female rats; sampling by decapitation under thiopental narcosis after 12 hr starvation period), was used for morphology study by conventional clinical diagnostic techniques at the Clinical Diagnostic Laboratory LABO-MED in Poznan. Biochemical blood analyses were conducted in the same laboratory using officially accepted standard clinical methods (chemiluminescence procedure on Immulite - DPC equipment). Precision of this technique is monitored by National Center of Quality of Diagnostic Medical Laboratories in Poland and the Laboratory is a participant of the International Quality Control RIQAS maintained by Randox Company. Histopathology of internal organs was determined at Pathomorphology Department, Medical University, Poznan and proximate analyses of skeletal muscles and bones were conducted at the Animal Feed and Nutrition Laboratory, Agricultural University, Poznan according to the standard AOAC procedure.

### Statistical analyses

Data were expressed as mean (±SEM) where applicable. Statistical analysis was performed by the Student’s t-test with the difference considered significant at *P*<0.05 and highly significant at *P*<0.01 and *P*<0.001).

## RESULTS

### Short-term study protocol (28 days)

**Body Weight**: The effect of administration of Maca-GO at levels 0.75 g and 7.5 g per kg body weight to male and female rats during the 28 day experimental period on the growth of animals is summarized in Table 2. Maca-GO reduced body weight (*P*<0.001) in male rats by 8.6% and 14.3% respectively in relation to their initial weight at the start of the Trial and as compared to the control group, which slightly gained weight during the same time period. No weight gains were recorded in all the female groups (control and Maca-GO).

**Table 2:** TRIAL I: Changes in body weight of male and female rats during short-term (28 days) administration of Maca-GO at two levels of 0.75 g and 7.5 g per kg body weight^a^

Oral dose of Maca-GO		0.75 g/kg			7.5 g/kg		
Group	Initial weight (g)	Final weight (g)	Change in the body weight (%)	Significance level	Final weight (g)	Change in the body weight (%)	Significance level

**Male**							
Control group	340 ± 7.3	350 ± 6.4	+2.9	*P*=0.27	350 ± 6.4	+2.9	*P*=0.27
Maca-GO group	350 ± 7.4	320 ± 6.6	-8.6	*P*<0.001	300 ± 8.9	-14.3	*P*<0.001
**Female**							
Control group	250 ± 6.3	250 ± 6.3	0	*P*=1.00	250 ± 6.3	0	*P*=1.00
Maca-GO group	250 ± 6.7	250 ± 8.9	0	*P*=1.00	250 ± 8.9	0	*P*=0.70

a Values in this and subsequent tables represent average of 10 animals per group of each males and females together with accompanied. Standard Error of Mean (±), together with the recorded absolute value for the calculated significance level of differences between the control and two experimental groups. Statistically-significant differences were considered as those below the *P*<0.05 and highly-significant changes below the *P*<0.01 level.

**Blood Morphology:** Due to observed similar pattern of recorded blood constituents in male and female rats, their individual morphology results were presented in Table 3 as combined male and female groups of rats receiving two doses of Maca-GO. During the 28 days of administration of the 0.75 g/kg Maca-GO dose resulted in statistically high significant increase (*P*<0.001) in white blood cell and lymphocyte counts as compared to control animals. There were no statistically significant (*P*<0.05) differences observed between groups in other morphological characteristics of blood. Similarly, Maca-GO applied at the 10 times higher dose (7.5 g/kg) resulted in a significant increase (*P*<0.05) in lymphocytes count, relative red cell decomposition index as well as in an average volume of red blood cells. There were no differences in other indices of morphological blood characteristics.

**Table 3:** TRIAL I: Morphology of blood in rats after 28 days administration of 0.75 g and 7.5 g of gelatinised Maca-GO per kg body weight of rats (values for male and female rats combined)

Oral dose of Maca-GO		0.75 g/kg		7.5 g/kg	
Blood morphology	Control Group	Maca-GO Group	Significance level	Maca-GO Group	Significance level

White Blood Cells - WBC (in 1 μl)	4.3 ± 0.9	7.6 ± 2.2	*p*<0.006	7.5 ± 3.5	*p*=0.06
Red blood Cells - RBC (w 1 μl)	6.6 ± 0.4	6.4 ± 0.5	*p*=0.51	5.8 ± 0.8	*p*=0.06
Haemoglobin - HGB (g/dl)	13.3 ± 0.8	12.8 ± 0.6	*p*=0.24	11.7 ± 1.6	*p*=0.06
Haematocrit - HCT (%)	34.2 ± 1.9	32.7 ± 2.1	*p*=0.25	33.8 ± 4.9	*p*=0.89
Average Volume of Red Blood Cell - MCV (fl)	51.8 ± 2.2	51.2 ± 2.0	*p*=0.60	58.8 ± 6.3	*p*<0.03
Average weight of haemoglobin in red blood cell - MCH (pg)	20.2 ± 0.9	20.0 ± 0.8	*p*=0.69	20.2 ± 0.5	*p*=0.93
Average concentration of haemoglobin in red blood cell - MCHC (g/dl)	38.9 ± 0.9	39.1 ± 0.8	*p*=0.74	34.8 ± 4.9	*p*=0.07
Platelets count - PLT (w 1 μl)	807 ± 143	903 ± 64	*p*=0.16	754 ± 190	*p*=0.61
Relative Lymphocyte count - LYMPH% (%)	76.9 ± 5.52	73.9 ± 1.83	*p*=0.22	73.0 ± 8.75	*p*=0.39
Lymphocyte count - LYMPH (1 μl)	3.3 ± 0.5	5.6 ± 1.6	*p*<0.006	5.3 ± 1.9	*p*<0.04
Red Cell Decomposition - RDW-CV (%)	12.8 ± 0.7	13.3 ± 0.7	*p*=0.22	20.5 ± 6.4	*p*<0.02
Platelets Index - PDW (fl)	17.1 ± 0.9	16.8 ± 0.5	*p*=0.47	16.7 ± 0.4	*p*=0.37
Average size of Platelets MPV (fl)	6.2 ± 0.5	5.7 ± 0.3	*p*=0.08	6.0 ± 0.3	*p*=0.69

**Biochemical analyses of blood serum:** Results of biochemical analyses of blood serum in rats administered Maca-GO at levels 0.75 g/kg and 7.5 g/kg during 28 days experimental period is given in Table 4. Maca-GO at level 0.75 g/kg resulted in statistically high significant decrease in serum cortisol level (*P*<0.001) and a noticeable (although statistically-not confirmed) increase in blood glucose level (from 83 do 111 mg/dl). The remaining blood indices in Maca-GO group did not showed statistically-significant differences in recorded values as compared to the control group.

**Table 4:** TRIAL I: Blood biochemistry in rats after oral administration of 0.75 g and 7.5 g of Maca-GO per kg body weight during 28 day period (values for male and female rats combined)

Oral dose of Maca-GO		0.75 g/kg		7.5 g/kg	
Biochemical blood serum indices	Control group	Maca-GO Group	Significance level	Maca-GO Group	Significance level

Glucose (mg/dl)	83 ± 21	111 ± 35	*p*=0.13	125 ± 44	*p*=0.07
Total Cholesterol (mg/dl)	73 ± 10	74 ± 16	*p*=0.85	66 ± 21	*p*=0.40
HDL - Cholesterol (mg/dl)	30 ± 10	27 ± 3	*p*=0.46	34 ± 2	*p*=0.30
LDL - Cholesterol (mg/dl)	7 ± 1	7 ± 1	*p*=1.00	7 ± 1	*p*=1.00
Triglycerides (mg/dl)	49 ± 17	32 ± 15	*p*=0.11	31 ± 7	*p*<0.04
Sodium (mol/l)	132 ± 13	140 ± 3	*p*=0.15	140 ± 3	*p*=0.14
Potassium (mmol/l)	5.8 ± 2.3	5.0 ± 0.7	*p*=0.43	5.1 ± 1.9	*p*=0.59
Iron (μg/dl)	227 ± 44	197 ± 50	*p*=0.29	209 ± 65	*p*=0.57
Cortisol (nmol/l)	5.4 ± 2.0	2.5 ± 0.8	*p*<0.01	2.5 ± 0.8	*p*<0.01
ACTH (pg/ml)	72 ± 112	144 ± 187	*p*=0.47	45 ± 30	*p*=0.58
TSH (μIU/ml)	0.16 ± 0.1	0.13 ± 0.1	*p*=0.42	0.19 ± 0.1	*p*=0.47
PSA (ng/ml) - (male only)	<0.04	<0.04	-	<0.04	-
Progesterone (ng/ml) - (female only)	26.2 ± 16.7	24.8 ± 8.8	*p*=0.90	3.2 ± 0.46	*p*=0.08
Estradiol (pg/ml) - (female only)	34.6 ± 17.8	40.4 ± 33.0	*p*=0.80	136.3 ± 21.1	*p*<0.003
LH (mIU/ml) - (female only)	0.17 ± 0.10	0.11 ± 0.01	*p*=0.25	<0.10	*p*=0.20
FSH (mIU/ml) - (female only)	<0.10^a^	<0.10^a^	-	<0.10	-
Prolactin (ng/ml) - (female only)	<0.50^a^	<0.50^a^	-	<0.50	-

a Values below the detection level.

Administration of 7.5 g Maca-GO to rats resulted in statistically significant decrease in serum cortisol (*P*<0.01) and triglycerides (*P*<0.05) levels, while estradiol concentration significantly increased (*P*<0.01). Also, noticeable (50%) but statistically not significant (*P*>0.05) increase in glucose level was observed, with a simultaneous 12% decrease in progesterone level. There were no significant differences between groups in the remaining blood indices.

**Histopathology:** After 28 days oral administration of the 0.75 g of Maca-GO to both groups of rats there were no changes recorded which could be attributed to Maca-GO treatment. All rats in both groups had histological characteristics of assessed internal organs within the picture considered as normal for animals at their stage of growth (Table 5).

**Table 5:** TRIAL I: Description of histopatological examination of internal organ in male and female rats after oral administration of 0.75 g and 7.5 g Maca-GO per kg body weight during 28 day period

Internal organ	Description of histopatological picture 0.75 g/kg	Description of histopatological picture 7.5 g/kg

Control Group		
Male		
Liver	Normal – No changes detected^a^	Normal – No changes detected
spleen	Normal – No changes detected	Normal – No changes detected
kidneys	Normal – No changes detected	Normal – No changes detected
prostate	Local enlargements of cells	Local enlargements of cells
seminal duct	Normal – No changes detected	Normal – No changes detected
urinary tract	Normal – No changes detected	Normal – No changes detected
testes	lumen of ducts slightly widened; epithelium slightly thinned	lumen of ducts slightly widened; epithelium slightly thinned
Female		
Liver	Normal – No changes detected	Normal – No changes detected
pancreas	Normal – No changes detected	Normal – No changes detected
kidneys	chronically inflamed blood vessels	slightly enlarged blood vessels with chronic inflammations; single eozynophiles present
uterus	severe chronic inflammations; numerous eozynophiles present	Normal – No changes detected
Maca-GO Group		
Male		
Liver	N/D	Normal – No changes detected
pancreas	Normal – No changes detected	Normal – No changes detected
spleen	Normal – No changes detected	Normal – No changes detected
kidneys	chronically inflamed blood vessels	Normal – No changes detected
prostate	slightly inflamed and enlarged cells	local slight inflammations of cells
seminal duct	Normal – No changes detected	Normal – No changes detected
urinary tract	Normal – No changes detected	Normal – No changes detected
Female		
liver	N/D	Normal – No changes detected
pancreas	N/D	Normal – No changes detected
spleen	N/D	Normal – No changes detected
kidneys	N/D	Normal – No changes detected
uterus	N/D	Normal – No changes detected

a Normal – No changes detected, No histopathological changes detected in microscopic examination–picture of individual slides considered as “normal”; N/D, Not determined.

Similarly as at the lower level (0.75 g/kg) of Maca-GO inclusion during 28 days trial, rats receiving a 7.5 g/kg dose of Maca GO showed no visible effect in histopathology of internal organs as compared to animals in corresponding control groups. In both, control and Maca-GO groups of male rats there were slight enlargement of cells in prostate, and in female group of control rats uterus showed enlargement in diameter of ducts and thinning of endometrium of uterus, the changes, which were not observed in female rats in the Maca-GO group. Histo-pathologic examination of the remaining organs in male rats (liver, spleen, pancreas, kidneys, seminal vessel urinary tract and testes) and female rats (liver, pancreas, spleen and kidneys), showed no visible changes which could be attributed to administered Maca-GO at the 7.5 g/kg level. The remaining internal organs of male and female rats in both groups exhibited histological characteristic considered normal for animals of the appropriate gender and at the stage of their physiological development.

### Sub-chronic study protocol (90 days)

**Body Weight:** In all four groups of both, male and female rats used in this Trial (II), there were statistically-significant (*P*<0.01) differences in weight gains recorded (Table 6) as a result of 90 day administration of Maca-GO at both levels 0.75 g/kg and 7.5 g/kg, with female gaining much less than male rats. Administering Maca-GO to female rats at both doses, lowered their weight gains in relation to the female control (8% gain against the 25% gain in the control group), while male group had also lower gains but the difference between the test and control groups was much lower (14%-17% against 20% respectively).

**Table 6:** TRIAL II: Changes in body weight of male and female rats during long-term (90 days) administration of gelatinised Maca-GO at two levels of 0.75 g and 7.5 g per kg bod weight of rats

Oral dose of Maca-GO		0.75 g/kg			7.5 g/kg		
Group	Initial weight	Final weight	Change in the	Significance	Final weight	Change in the	Significance
	(g)	(g)	body weight (%)	level	(g)	body weight (%)	level

Male							
Control group	340 ± 7.3	350 ± 6.4	+2.9	*p*<0.001	420 ± 8.9	+20.0	*p*<0.001
Maca-GO group	350 ± 7.4	320 ± 6.6	-8.6	*p*<0.001	400 ± 2.6	+14.3	*p*<0.001
Female							
Control group	250 ± 6.3	250 ± 6.3	0	*p*<0.001	300 ± 8.8	+25.0	*p*<0.001
Maca-GO group	250 ± 6.7	250 ± 8.9	0	*p*<0.002	270 ± 6.1	+8.0	*p*<0.002

**Blood Morphology:** There were no statistically significant (*P*>0.05) differences in morphological characteristics of blood of rats receiving for 90 days Maca-GO at both, 0.75 g/kg and 7.5 g/kg levels (Table 7).

**Table 7:** TRIAL II: Morphology of blood during long-term (90 days) administration of Maca-GO at levels of 0.75 g/kg and 7.5 g/kg body weight of male and female rats (values for male and female rats combined)

Oral dose of Maca-GO		0.75 g/kg		7.5 g/kg	
Blood morphology	Control Group	Maca-GO Group	Significance level	Maca-GO Group	Significance level

White Blood Cells - WBC (in 1 μl)	6.1 ± 2.2	6.5 ± 3.6	*p*=0.66	5.9 ± 2.2	*p*=0.85
Red blood Cells - RBC (w 1 μl)	6.5 ± 0.8	6.1 ± 1.3	*p*=0.29	6.2 ± 0.8	*p*=0.35
Haemoglobin - HGB (g/dl)	13.4 ± 0.9	12.3 ± 2.3	*p*=0.07	12.9 ± 1.3	*p*=0.15
Haematocrit - HCT (%)	35.2 ± 2.9	33.2 ± 3.9	*p*=0.11	34.1 ± 3.6	*p*=0.34
Average Volume of Red Blood Cell - MCV (fl)	54.8 ± 4.8	56.3 ± 7.9	*p*=0.52	55.2 ± 2.8	*p*=0.77
Average weight of haemoglobin in red blood cell - MCH (pg)	20.9 ± 1.8	20.4 ± 1.8	*p*=0.42	20.9 ± 1.6	*p*=0.93
Average concentration of haemoglobin in red blood cell - MCHC (g/dl)	38.2 ± 1.8	36.7 ± 4.4	*p*=0.22	37.8 ± 1.9	*p*=0.55
Platelets count - PLT (w 1 μl)	778 ± 86	730 ± 123	*p*=0.22	721 ± 135	*p*=0.17
Relative Lymphocyte count - LYMPH% (%)	76.3 ± 10.7	73.1 ± 3.72	*p*=0.29	74.4 ± 4.44	*p*=0.51
Lymphocyte count - LYMPH (1 μl)	4.5 ± 1.4	4.8 ± 2.6	*p*=0.74	4.4 ± 1.4	*p*=0.74
Red Cell Decomposition - RDW-CV (%)	15.2 ± 2.7	16.3 ± 3.1	*p*=0.29	14.6 ± 1.8	*p*=0.51
Platelets Index - PDW (fl)	17.4 ± 0.9	17.5 ± 0.9	*p*=0.72	17.9 ± 1.1	*p*=0.17
Average size of Platelets MPV (fl)	6.5 ± 0.6	6.3 ± 0.7	*p*=0.46	6.8 ± 0.9	*p*=0.26

**Biochemical analyses of blood serum:** Similarly to the short-term trial, a long-term administration of Maca-GO at 0.75 g/kg level resulted in a significant decrease in serum cortisol (*P*<0.05) only (Table 8). There was a noticeable, but statistically not significant, increase (*P*>0.05) in glucose and progesterone levels, and a decrease in triglycerides and ACTH levels (*P*>0.05). There were no significant differences (*P*> 0.05) detected between the groups in the remaining biochemical blood plasma characteristics.

**Table 8:** TRIAL II: Biochemistry of blood in after long-term (90 days) oral administration of 0.75 g and 7.5 g Maca-GO per kg body weight of male and female rats (combined male and female results)

Oral dose of Maca-GO		0.75 g/kg		7.5 g/kg	
Biochemical blood serum indices	Control group	Maca-GO Group	Significance level	Maca-GO Group	Significance level

Glucose (mg/dl)	89 ± 30	106 ± 19	*p*=0.08	112 ± 17	*p*<0.01
Total Cholesterol (mg/dl)	77 ± 15	75 ± 15	*p*=0.75	71 ± 19	*p*=0.29
HDL - Cholesterol (mg/dl)	32 ± 6	32 ± 7	*p*=0.87	30 ± 9	*p*=0.42
LDL - Cholesterol (mg/dl)	14 ± 4	12 ± 3	*p*=0.06	13 ± 5	*p*=0.31
Triglycerides (mg/dl)	59 ± 22	61 ± 15	*p*=0.78	58 ± 16	*p*=0.89
Sodium (mol/l)	138 ± 4	136 ± 3	*p*=0.15	138 ± 4	*p*=0.77
Potassium (mmol/l)	4.8 ± 0.9	4.3 ± 0.8	*p*=0.13	3.8 ± 0.4	*p*<0.0003
Iron (μg/dl)	113 ± 23	136 ± 42	*p*=0.08	124 ± 51	*p*=0.45
Cortisol (nmol/l)	4.7 ± 2.1	3.0 ± 1.8	*p*<0.03	3.9 ± 2.2	*p*=0.38
ACTH (pg/ml)	108 ± 105	71 ± 85	*p*=0.31	74 ± 55	*p*=0.26
TSH (μIU/ml)	0.23 ± 0.1	0.19 ± 0.1	*p*=0.35	0.24 ± 0.2	*p*=0.83
PSA (ng/ml) – (male only)	<0.04	<0.04	-a	<0.04	-a
Progesterone (ng/ml) – (female only)	5.6 ± 3.1	9.1 ± 8.3	*p*=0.33	10.6 ± 5.8	*p*<0.04
Estradiol (pg/ml) – (female only)	51.5 ± 20.9	50.3 ± 17.2	*p*=0.91	46.6 ± 20.1	*p*=0.90
LH (mIU/ml) - (female only)	0.10 ± 0.01	0.11 ± 0.03	*p*=0.61	0.10 ± 0.01	*p*=0.33
FSH (mIU/ml) - (female only)	<0.10	<0.10	-a	<0.10	-a
Prolactin (ng/ml) - (female only)	<0.50	<0.50	-a	<0.50	-a

a Values below the detection level.

Long-term (90 days) administration of Maca-GO (7.5 g/kg) to female rats, resulted in statistically high significant increase in blood glucose concentration (*P*<0.01) and significant increase in progesterone level (*P*<0.05), with statistically not significant but noticeable decrease in both Cortisol and ACTH levels as compared to the control group.

**Histopathology of internal organs:** Administering rats with a 7.5 g/kg dose of Maca GO for the extended period of time had no effect on histopathology of internal organs as compared to animals in a control group. All the assessed samples, exhibited histological characteristic considered normal for animals at their stage of physiological development and gender (Table 9).

**Table 9:** TRIAL II: Description of histopatological picture of internal organs in male and female rats after oral administration of 0.75 g and 7.5 g Maca-GO per kg body weight during 90 day period

Group/Sex/Organ	Description of histopatological picture 0.75 g/kg	Description of histopatological picture 7.5 g/kg

Control Group		
Male		
Liver	single inflammations with lymphocytes present but cells appearance normal	single concentrated intracell inflammations within normal cell structure
pancreas	Normal – No changes detected^a^	Normal – No changes detected
spleen	Normal –single hemosyderyne deposits	Normal –single hemosyderyne deposits
kidneys	Sporadic cysts and inflammations of cells in cortical layer of cells	Sporadic cysts and inflammations of cells in cortical layer of cells
testes	Normal – No changes detected	Normal – No changes detected
Female		
Liver	Single concentrated cell inflammations but liver cells normal	Single concentrated cell inflammations within normal cell structure
pancreas	Normal – No changes detected	Normal – No changes detected
spleen	Cells inflamed with hemosyderine deposits	Cells inflamed with hemosyderine deposits
uterus	abscesses embedded in the epithelial layer	single abscesses in the epithelium
Maca-GO Group		
Male		
Liver	separate concentrations of lymphocytes	Normal – No changes detected
pancreas	Normal – No changes detected	Normal – No changes detected
spleen	Normal – traces of fresh vasculature	N/D
kidneys	local cysts in nephrite layer with a homogenous eozynophyllic contents	N/D
testes	Normal – No changes detected	Normal – No changes detected
Female		
Liver	Normal – No changes detected	separate concentrations of lymphocytes within normal cell structure
pancreas	Normal – No changes detected	N/D
uterus	distorted cell structure of the wall	N/D
kidneys	single concentrations of lymphocytes in cortical layer and enlarged lumen of nephrites filled with homogenous eosynophyllic substance	single concentrations of lymphocytes in cortical layer and enlarged lumen of nephrites filled with homogenous eosynophyllic substance

a Normal – No changes detected, No histopathological changes detected in microscopic examination–picture of individual slides considered as “normal”; N/D, Not determined.

**Chemical composition of muscle and bone tissues:** Results of chemical analyses from pooled homogenates of skeletal muscles and bones obtained from dissected hind legs of rats exposed for 90 days to two levels of Maca-GO (0.75 g/kg and 7.5 g/kg) are summarised in Table 10. Irrespective of the level of Maca-GO administered to male rats, there was a substantial reduction (17% to 19%) in total lipid contents determined in homogenates of muscle and bones. However, there was a reverse effect observed in female rats showing an increase in tissue fat content by 27% to 30% in comparison to control female rats. While there were no distinctive differences in dry matter and protein contents in homogenates, there was an increase in ash level determined in male rats receiving Maca-GO at the 0.75 g/kg level only and this was accompanied by an increase in both Calcium and Phosphorus contents (11% and 19% respectively). There was a lesser increase in Calcium and Phosphorus concentrations in tissue homogenates observed in female groups of rats, however, all the recorded values at both levels of Maca-GO administration were above the corresponding levels determined in corresponding control groups of female rats.

**Table 10:** TRIAL II: Chemical analyses of homogenates from skeletal muscles and bones obtained from hind legs of male (M) and female (F) rats after a long-term (90 days) administration of two doses (0.75 g/kg and 7.5 g/kg body weight) of gelatinised Maca-GO

Group	Dry Matter (%)		Ash (%)		Crude Protein (N × 6.25) (%)		Total Fat (%)		Calcium (%)		Phosphorus (%)	
	M	F	M	F	M	F	M	F	M	F	M	F

Control Group	36.60	35.61	7.16	7.35	23.21	23.61	6.00	4.02	1.94	1.97	1.26	1.29
Maca-GO (0.75 g/kg)	37.20	36.88	7.85	7.38	24.26	23.03	4.91	6.17	2.15	2.09	1.51	1.32
Maca-GO (7.5 g/kg)	35.83	36.38	6.83	7.55	23.98	23.48	4.98	5.33	2.02	2.05	1.38	1.42

### TOXICITY (LD50) for Maca-GO

All animals survived the test without any adverse effects noticed on the basis of abnormal behavior and histopathology of internal organs (liver, spleen, pancreas, testis and ovaries). On the basis of the obtained results, adopting recommendations by Hodge & Sterner, the 7.5 g Maca-GO per kg body weight (the highest dose applied in this study) was determined as LD_50_ for Maca-GO, established in a standard bioassay to have no toxic effects on test animals and upto this level considered to be safe for oral administration in therapeutic and dietary preparations. The above dose is considerably higher than the 2 g/kg limit determined by the OECD (14) as non toxic and safe for use as dietary supplements.

Table 1 shows the clinical features for the EH patients and the NT controls. The SBP and DBP were significantly higher in the EH group as compared to that seen in the NT group. Age, body mass index (BMI), pulse rate, serum concentrations of creatinine and uric acid, and plasma concentrations of total cholesterol did not significantly differ between the two groups.

We performed an association study using 4 SNPs. Table 2 shows the genotype distributions of the 4 SNP variants and allelic frequencies. The overall distribution of the genotypes did not significantly differ between the EH and NT groups. However, there was a difference noted for the allelic frequency of the SNP rs2290105 between women in the EH and NT groups (*p* value=0.0409), with the allelic frequency of the minor allele in the EH group higher than that seen for the NT group.

The haplotype-based case-control study also documented significant differences between the women of the EH and NT groups (Table 3). There were significant differences for the results of the A-T-A-C haplotype, with this haplotype higher in the EH (42.6%) than in the NT group (28.8%).

## DISCUSSIONS

It has been generally-accepted, that Maca exhibits specific – yet unresolved to the present days - endocrine effect, which, has been demonstrated in diverse areas of its biological action from being an energizing plant (11), stimulating reproductive functions (7-9) and balancing hormones (11, 12) as well as alleviating physical, physiological and psychological discomfort associated with menopause in women (1, 5, 6). However, until convincing proof is found as to the individual active compound, biochemically-identified as a key active Maca root component, or specific group of them, responsible for therapeutic functionality of Maca root, the authors decided to use in their study and recommend to use further in therapeutic practice, the entire root in its entity with its cohesive complexity considered as compositionally-unaltered herb with its historically-acknowledged and traditionally-unquestioned medicinal properties. The complexity of components present in Maca root powder such as sterols (campesterol, stigmasterol and beta-sitosterol), polyunsaturated acids and their amides, called “macaenes” and “macamides” (9, 11), aromatic glucosinolates (16) and several alkaloids – yet to be characterized , through their complex synergistic and/or interactive action amongst them, will eventually provide an answer to physiological action of specific doses of standardized Maca preparations recommended for prophylactic and/or specific therapeutic effect for men and women.

Since the first report of Chacon on medicinal properties of Maca root (10), it has been generally accepted and confirmed that this plant doesn’t contain plant estrogens or any other phyto-hormones (15, 17-19), but through plant sterols, stimulate endocrine system helping to maintain hormonal balance (10) in a way that is not yet well understood (6, 13). According to Muller (5), these sterols are used by the body with the help of the pituitary to improve adrenal function, ovarian and testicular function, as well as the functioning of the thyroid and the pancreas, and the pineal gland. Multi-functional effect of Maca on endocrine relationships may also explain reported in the literature, its positive influence on stimulation of endocrine glands in regulation of hormonal balances in the body (3, 20).

Present study were conducted on growing male and female rats, which, in addition to blood morphology, biochemistry and hormonal changes, allowed for an observation of growth patterns during short-term and longer-period of Maca-GO administration at two application levels. It appears that Maca applied to male rats at high dose (7.5 g/kg) during a short-term trial, significantly reduced body weights, while female rats have maintained their weight unchanged. However, when Maca was applied during longer period of time (90 days), female rats showed lesser body weight gains in relation to control female rats and as compared to male rats. The results indicate that Maca-GO administration induces body weight reducing action, howver, affecting male and female rats in a different way, and again differently in a short- and a longer-term study. This positive effect of Maca-GO on reduction of body weight in male and female rats could not be explained by lowering in TSH content in the blood, which usually leads to slowing down body metabolism, hence reducing the weight gains of rats.

In the Trial I, where a slight increase in white cell and lymphocyte counts and lowering hemoglobin content was recorded, may indicate an overall defensive blood morphology status in animals due to some factors other than Maca, such as may be observed when animals are fighting some mild infection. Signs of mild changes observed in histopathology of internal organs dissected from rats after a short-term trial, at lower (0.75 g/kg) Maca-GO administration level may support this assumption. Longer-term Maca-Go use, have not produced any significant changes in morphology nor in histopathology picture of rats.

Observed in the Trial I effect of 7.5 g/kg level of Maca-GO on an increased Estradiol level was accompanied by lowering in progesterone level which may indicate that blood sampling for analyses was done after the ovulation. In this trial, due to difficulties in precise detection of a menstrual cycle stage at the time of blood sampling from female rats (mainly due to their peculiar ability to spontaneous ovulations), the status of ovulation cycle was not determined. On the other hand, in a longer term trial, the level of Maca-GO application had no effect on estradiol level but simultaneous increase of progesterone contents could keep estradiol at uniformly moderate level at both Maca-GO application doses. The observed relationship between progesterone and estradiol are in accord with observations made by Lucille (21) in clinical practice, emphasizing the balance between progesterone, estradiol and thyroid function as one of the key factors in female maintaining hormonal balance during the reproduction years and in menopause. It is a function of progesterone to control estradiol and negative effects of its dominance as well as to support thyroid function in maintaining growth, healthy bone metabolism and balancing psychological equilibrium in females during and after their reproductive stage.

It was an interesting to observe a positive significant effect of Maca-GO on blood cortisol reduction in both short-and long-term trial, with more pronounced effect visible in Trial I (28 days). This may indicate positive effect of Maca-GO on lowering susceptibility of rats to stress factors and its sedative effect on laboratory animals, the properties also reported by Lopez Fondo *et al*. (22). Observed in Trial II, a long-term effect of both, low and high doses of Maca-GO administration on maintaining, or slightly reducing Estradiol level, at simultaneous increase in Progesterone in rats, opens a new important avenue for application of Maca-GO during perimenopausal phase in women, since at this stage of women life, there is a tendency to gradually increase Estradiol level due to decline in secretion of Progesterone (23). Gradually increasing blood estradiol level in perimenopausal stage leads to development of depression (24) in females at the perimenopause stage. It appears that an extended application of low doses of Maca-GO may slow down, delay or even prevent depression and other unpleasant symptoms which are manifested prior to and/or during the menopause (1, 5, 6). In a preliminary study on ovariectomised rats (25) it has been suggested that Maca-GO posessess anti-depressant-like and sedative, but not anxiolytic effects as measured in locomotor activity test, Porsolt and anxiolytic activity tests which corresponded with significant lowering in Cortisol and ACTH concentrations, leading to conclusion that Maca-GO could have value in treatment of some depressive symptoms during perimenopausal period. The follow-up study (26) on the same samples of Maca-GO as used in this study, when tested against Fluoxentine, a known antidepressant agent, confirmed the above assumptions showing that Maca-GO possesses typical antidepressant–like characteristics.

After Maca-GO administration to ovariectomised rats, both blood Cortisol and ACTH as well as spontaneous activity and immobility time (Porsolt test) were significantly (*P*<0.05) reduced, while Fluoxetine induced anti-depressive effect in control, non-ovariectomised animals only, without affecting ovariectomised rats, with one exception, that, Fluoxetine increased the blood Cortisol only in non-ovariectomised rats without significantly affecting ACTH and spontaneous activity test values. This led to conclusion that antidepressive action of Maca-GO is based on different mode of action in non- and ovariectomised rats as compared to the antidepressive effect of Fluoxentine on both groups of female rats. The above results from a model laboratory tests (25, 26) in conjunction with the results recorded in this study on male and non-ovariectomised female rats suggest that active phyto components present in Maca-GO act in a specific way on release of body steroids or affecting the hypothalamo-pituitary-ovarian axis in female rats (or -testes in males) resulting in triggering similar, but other than serotoninergic response mechanisms as observed in anti-depressive action of Fluoxentine on rats. This assumption however, needs to be tested clinically.

Maca-GO had positive effect on increased level of glucose in the blood of rats at both levels of application, however only in long-term Trial II, the high dose of Maca-GO administered to rats resulted in statistically significant rise in blood glucose level. This may explain reported energizing effect of Maca and its use as an energizing dietary supplement for sport people and those whose lifestyle requires energy reserves for intensive physical activity (3, 24, 27, 28). Obsrved in this study (Tables 4 and 7) increased glucose level in groups of rats receiving Maca-GO contradicts results reported by Lopez-Fernando (22) who observed hypoglycemic effect of methanolic extract of maca on stressed animals. Possible explanation of the discrepancy in results obtained in these two studies, may be due to response of non-stressed animals used in the present study, as opposed to stressed one in Lopez-Fernando’s experiment and/or due to carbohydrate fraction presence in Maca-GO, while this particular fraction, as insoluble in methanol, was not present in the extract administered to stressed animals.

There was a trend observed in this study across the all groups receiving Maca-GO that red blood cells, haematocrit, haemoglobin and other red cell characteristics were lower in recorded values as compared to the corresponding control groups of rats. This was associated with lowering in blood iron concentration at lower doses of Maca-GO, while at higher doses in short-term Trial and during an extended period had no such visible effect. This may indicate Maca-Go playing a role in modulating availability of dietary iron, the fact which could be explained by alteration in absorption of iron from the intestines (29), or by possible chelating effect of Maca-GO on dietary iron, without discounting the fact of lesser diet being consumed by rats after forced feeding them with Maca-GO. Overall haematology indices were within the norms considered as normal for rats (30).

This study to certain degree confirmed observation of Muller (6) that Maca, depending on the level and the length of its intake may act either as an adaptogenic herb displaying “stimulating” or “balancing” effect. However, higher doses during extended period of administration were not necessary more effective in inducing changes in blood indices and other tissue measurements taken in this study.

Observations made in this laboratory study justify further clinical research on use of Maca in peri-menopausal women and on physically-active people and sportsmen, this in order to assess effectiveness of Maca-GO as an energizing, non-hormonal, therapeutic supplement and as a potential substitute to HRT programs. Result obtained in earlier pilot study on use of pre-gelatinised Maca in early postmenopausal women (31), confirmed that through balancing hormones in the body, Maca-GO helped women to reduce discomfort which they experienced in post-menopausal stage. On the basis of results obtained in this laboratory study on rats, it is reasonable to suppose that Maca-GO may help women to reduce discomfort experienced not only in post-menopause, but also well before onset of menopause and during entering menopausal stage, which may simultaneously assist in restricting weight increase, lowering triglycerides in blood plasma and an increase in calcium and phosphorus deposition in bone and muscle tissues.
